# Numerical Simulation of Tube Manufacturing Consisting of Roll Forming and High-Frequency Induction Welding

**DOI:** 10.3390/ma15031270

**Published:** 2022-02-08

**Authors:** Christian Egger, Marco Lüchinger, Michael Schreiner, Wolfgang Tillmann

**Affiliations:** 1 Institute für Computational Engineering (ICE), Eastern Switzerland University of Applied Sciences, 9471 SG Buchs, Switzerland; marco.luechinger@ost.ch (M.L.); michael.schreiner@ost.ch (M.S.); 2 Institute of Materials Engineering (LWT), Technical University of Dortmund, 44227 Dortmund, Germany; wolfgang.tillmann@udo.edu

**Keywords:** high-frequency induction welding, electromagnetic heating, phase transformation, finite element method, multiphysics simulation, dual mesh method

## Abstract

This paper presents a fully coupled three-dimensional finite element model for the simulation of a tube manufacturing process consisting of roll forming and high-frequency induction welding. The multiphysics model is based on the dual mesh method. Thus, the electromagnetic field, the temperature field, the elasto-plastic deformation of the weld bead, and the phase transformations within the material can be simulated for a moving tube without remeshing. A comparison with measurements shows that the geometry of the welded tube and the weld bead, the force on the squeeze rolls, the temperature along the band edges, and the hardness distribution within the heat-affected zone can be simulated realistically.

## 1. Introduction

The demand for tubes with high strength and ductility has grown in recent years, especially in the automotive industry. In order to reduce the weight of cars, tubes are often used to replace parts which formerly were made of solid material [1,2]. Currently, around 70% of steel tubes with a diameter of less than 300 mm are fabricated as longitudinal welded tubes [3]. In the tube manufacturing process, a steel band is formed into an open seam tube by roll forming. Then, the remaining slit is closed by welding. The most common welding technique is high-frequency induction (HFI) welding.

High strength steel tubes in general have a carbon equivalent of more than 0.45%. Such steel grades are easily hardenable and thus difficult to weld, since cold cracks can occur along with martensite formation [4]. In order to reliably process such steel grades, the HFI welding process has to be optimized. However, welding parameters are difficult to measure in the field. An established technique for studying complex, difficult-to-measure physical interactions is the finite element method (FEM). Whereas the simulation of roll forming is state of the art, only a few, simplified models for the HFI welding process exist.

The first numerical simulations of the roll forming process were performed by Masuda et al. [5]. Wen and Pick [6], Kiuchi et al. [7], and later Salmani et al. [8] used the FEM to improve the roll forming process regarding edge buckling, which makes welding impossible. Numerical models have often also been used for the comparison of roll forming strategies [9,10,11] and the investigation of process parameters [12,13,14]. Furthermore, Kim et al. [15] used the FEM to minimize the deformation of the band edge during roll forming. Many scientific publications have proven that the geometry of the open seam tube can be predicted correctly by means of FEM [16,17,18,19]. This also holds for residual stresses, which arise in the tube during roll forming, as shown by Bauer et al. [20].

The HFI welding process was long studied based on analytical expressions, for example, by calculating the current distribution within the tube [21,22,23]. The analytical methods were replaced by simple two-dimensional finite element (FE) models, which enabled the calculation of the current as well as the temperature distribution sequentially [24,25]. This means that there is no bidirectional coupling of the electromagnetic and the temperature field. The same holds for the simulation of Kim and Young [26], who presented a three-dimensional model for studying the temperature distribution within tubes. A bidirectional coupled electromagnetic-thermal simulation was first published by Nikanorov et al. [27,28]. Similar approaches have been used to investigate the influence of welding parameters such as current frequency, V-angle, or welding speed on the temperature distribution within the tube [29,30,31,32,33]. The elasto-plastic behavior of the band edges was first taken into account by Okabe et al. [34,35], who investigated the effect of the welding speed on the strain and stress distribution in the weld seam based on a two-dimensional FE model. A coupled three-dimensional model (electromagnetic-thermal-mechanical) was recently published by Ghaffarpour et al. [36] to study the weld bead formation. Although significant scientific work already has been done, no welding simulation takes into account the exact geometry of the band edges and the residual stresses within the steel band that arise during roll forming. Moreover, the movement of the tube as well as phase transformations within the material due to temperature changes were neglected in all of the mentioned publications.

In this paper, a three-dimensional FE model for a coupled electromagnetic-thermal-mechanical simulation of the tube manufacturing process consisting of roll forming and HFI welding is presented. Compared to other works, the welding simulation considers the exact geometry of the open seam tube, the residual stresses generated during roll forming, the movement of the tube, and phase transformations within the material caused by temperature changes. In order to validate the simulation, the geometry of the final tube and the weld bead, the force on the squeeze rolls, the temperature along the band edges, the phase types after welding, and the hardness distribution within the heat-affected zone were compared to measurements. It was shown that the mentioned quantities can be predicted realistically by means of FEM.

## 2. Materials and Methods

### 2.1. Longitudinal Tube Welding

**Figure 1:**
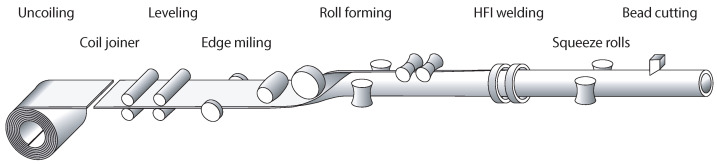
Schematic of the process chain for manufacturing longitudinal welded tubes.

Longitudinal welded tubes are formed from cold- or hot-rolled steel band, which is delivered as a coil. In order to enable a continuous forming and welding process, several coils are joined together. The process chain for manufacturing longitudinal welded tubes is shown schematically in Figure 1.

**Figure 2:**
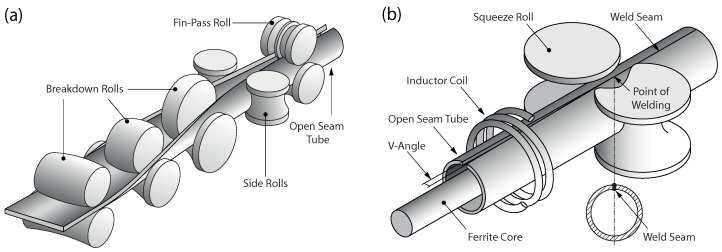
Detailed schematic of (**a**) roll forming and (**b**) HFI longitudinal tube welding.

After uncoiling, joining, and leveling, the band edges are milled to adjust the width of the band to the desired dimension. At the same time, damages to the band edges that could negatively affect the welding process are removed. Then, the steel band passes several roll forming stands with breakdown and fin-pass rolls, as shown in Figure 2a. Within the forming stands, the steel band is gradually formed into an open seam tube. Depending on the size and wall thickness of the tube, different forming strategies are used. The open seam tube is finally joined by welding. Because of the high speeds of up to 200 m/min in tube welding lines, HFI welding is typically used. The HFI welding process is shown schematically in Figure 2b. In HFI welding, the heat is generated by electromagnetic induction. For that purpose, an inductor coil encloses the open seam tube in front of the point of welding. The inductor coil is fed by a high-frequency generator with a frequency in the range of 50–500 kHz and a power of up to 1000 kW. With a ferrite core (impeder) placed inside of the tube, the generated electromagnetic field is concentrated in the region of the band edges. Due to the alternating electromagnetic field, a current is induced in the circumferential direction of the tube. Since the tube is open seam at the location of the inductor coil, the current flows to the point of welding and back in order to establish a closed circuit. Because of the skin effect and the proximity effect, the current flow remains restricted to the band edges. Thus, only the band edges are heated, with the highest temperature reached at the point of welding. Close to this point, the molten material is joined by squeeze rolls attached to the side of the tube. Due to the lateral pressure, molten material is squeezed out of the welding zone. This forms so called weld beads on the inside and outside of the tube, which are immediately removed by shaving. Finally, the continuously manufactured tube is cut to the desired length by a traveling saw.

As mentioned, only a small zone along the band edges is significantly heated during HFI welding, while the rest of the tube remains almost cold. Due to high temperature gradients and the thermal conductivity of the material, the heat dissipates quickly from the heat-affected zone. In steels with a carbon equivalent of >0.45%, this can lead to a hardening of the weld seam.

### 2.2. Mathematical Modeling and Material Data

**Figure 3:**
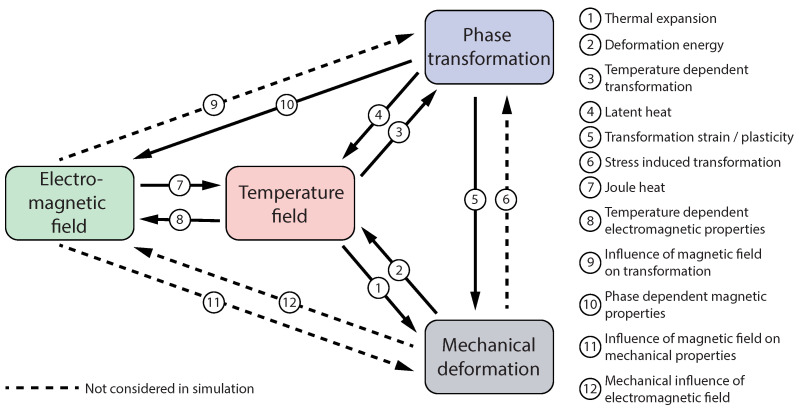
Schematic representation of the physical interactions during HFI welding.

In the following section, the governing and constitutive equations as well as the material data required for modeling electromagnetic-thermal-mechanical coupled problems are presented. An overview of how the electromagnetic field, the temperature field, phase transformations, and mechanical deformations interact is given in Figure 3.

#### 2.2.1. Electromagnetic Field

For a general time-varying electromagnetic field, the Maxwell’s equations [37] can be written in the differential form

(1) $$\nabla \times H=J+\frac{\partial D}{\partial t},$$

(2) ∇·B=0,

(3) $$\nabla \times E=-\frac{\partial B}{\partial t},$$

(4) $$\nabla \;\cdot \;D=\rho_{c}.$$

where B is the magnetic flux density, D the electric flux density, E the electric field intensity, H is the magnetic field intensity, J the current density, and $\rho_{c}$ the electric charge density. For a linear isotropic medium, the link between these field quantities is given by

(5) J=κ(T,Θ)E,

(6) $$B=\mu_{0}\;\mu_{r}\left(T,H,\Theta \right)\;H,$$

(7) $$D=\epsilon_{0}\;\epsilon_{r}\;E,$$

where κ is the electrical conductivity, $\mu_{0}$ the vacuum permeability, $\mu_{r}$ the relative magnetic permeability, $\epsilon_{0}$ the vacuum permittivity. and $\epsilon_{r}$ the relative permittivity of the material. Further, *T* is the temperature and Θ the volume fraction of a phase.

A vector magnetic potential A and a scalar potential *V* are now introduced, such that

(8) B=∇×A,

(9) $$E=-\frac{\partial A}{\partial t}-\nabla V.$$

Inserting (8) and (9) into the Maxwell’s equations and assuming a time-harmonic excitation with an angular frequency ω, the differential equations

(10) $$\frac{1}{\mu_{0}\;\mu_{r}\left(T,H,\Theta \right)}\left(\nabla \times \nabla \times A\right)+\kappa \left(T,\Theta \right)\left(\nabla V+i\omega A\right)=0,$$

(11) $$\nabla \;\cdot \;\left(\kappa \left(T,\Theta \right)\left(\nabla V+i\omega A\right)\right)=0,$$

which are solved by means of FEM, can be derived.

#### 2.2.2. Temperature Field

Based on Fourier’s law [38], the heat equation can be written in the form

(12) $$c_{p}\left(T,\Theta \right)\;\rho_{v}\left(T,\Theta \right)\;\frac{\partial T}{\partial t}-\nabla \;\cdot \;\left(k(T,\Theta )\nabla T\right)=Q,$$

where $c_{p}$ is the specific heat, $\rho_{v}$ the density, *T* the temperature, and *k* the thermal conductivity of the material. The heat source consisting of joule heating, the dissipation of elastic and plastic work, and the heat produced by phase transformations are described by

(13) $$Q=\kappa \omega^{2}{\left|A\right|}^{2}+T\frac{\partial \sigma }{\partial T}:{\dot{\varepsilon }}_{e}+q\sigma :{\dot{\varepsilon }}_{p}+\sum_{j=1}^{m}\Delta H_{j}\frac{d\zeta_{j}}{dt},$$

where σ is the stress tensor, ${\dot{\varepsilon }}_{e}$ the elastic strain rate tensor, and ${\dot{\varepsilon }}_{p}$ the plastic strain rate tensor. *q* is the fraction of plastic work transferred into heat. The enthalpy absorbed or released during a transition of phase *j* is described by $\Delta H_{j}$, while $\zeta_{j}$ is its volume fraction and *m* is the number of phases.

#### 2.2.3. Phase Transformation

For describing diffusion-controlled solid state transformations, the Johnson–Mehl–Avrami–Kolmogorov (JMAK) equation is used [39,40]. Thus, the phase transformations from ferrite or pearlite to austenite and from austenite to ferrite or pearlite are modeled with the equation

(14) $$\zeta \left(T,t,\Theta \right)=\zeta_{e}\left(T,\Theta \right)\left(1-\exp\left(-{\left(\frac{t}{\tau (T,\Theta )}\right)}^{n(T,\Theta )}\right)\right),$$

where ζ is the volume fraction of a phase Θ, $\zeta_{e}$ the equilibrium phase fraction, τ the time scale, and *n* the phase transition rate.

For describing diffusionless solid state transformations, the Koistinen–Marburger equation is used [41]. Consequently, the phase transformation from austenite to martensite is modeled with the equation

(15) $$\zeta \left(T\right)=\zeta_{e}\left(T\right)\left(1-\exp\left(\frac{M_{S}-T}{T_{0}}\right)\right),$$

where $M_{S}$ is the martensite start temperature and $T_{0}$ the reference temperature.

#### 2.2.4. Mechanical Deformation

Assuming small strains, the strain tensor

(16) $$\varepsilon =\varepsilon_{e}+\varepsilon_{p}+\varepsilon_{\mathrm{th}}+\varepsilon_{v}+\varepsilon_{\mathrm{tp}}$$

can be decomposed into an elastic, plastic, thermal, volume changing, and transformation-induced part. The elastic strain is described based on Hooke’s law

(17) $$\varepsilon_{e}=S\left(T,\Theta \right):\sigma ,$$

where S is the elastic compliance tensor. Assuming an associated flow rule

(18) $${\dot{\varepsilon }}_{p}=\lambda \frac{\partial f(\sigma ,\varphi ,\dot{\varphi },T,\Theta )}{\partial \sigma },$$

where *f* is the yield condition and λ is a scalar plastic multiplier, the plastic strain can be described by

(19) $$\varepsilon_{p}=\sqrt{\frac{2}{3}}\;\int_{0}^{t}∥{\dot{\varepsilon }}_{p}\left(\tau \right)∥\;d\tau .$$

Further, the thermal strain can be calculated with

(20) $$\varepsilon_{\mathrm{th}}=\alpha \left(T,\Theta \right)\left(T-T_{r}\right),$$

where α is the thermal expansion tensor and $T_{r}$ is the reference temperature. The volumetric change due to a phase transformation is described by

(21) $$\Delta \varepsilon_{v}=\sum_{j=1}^{m}\varepsilon_{v,j}\Delta \zeta_{j}$$

where $\varepsilon_{v,j}$ is the strain caused by the volume change of phase *j*. The transformation-induced plastic strain (TRIP) is calculated with

(22) $$\Delta \varepsilon_{\mathrm{tp}}=\left(\sum_{j=1}^{m}C_{\mathrm{tp},j}\Delta \zeta_{j}\right)\sigma$$

where $C_{\mathrm{tp},j}$ is the TRIP coefficient of phase *j*.

#### 2.2.5. Material

**Figure 4:**
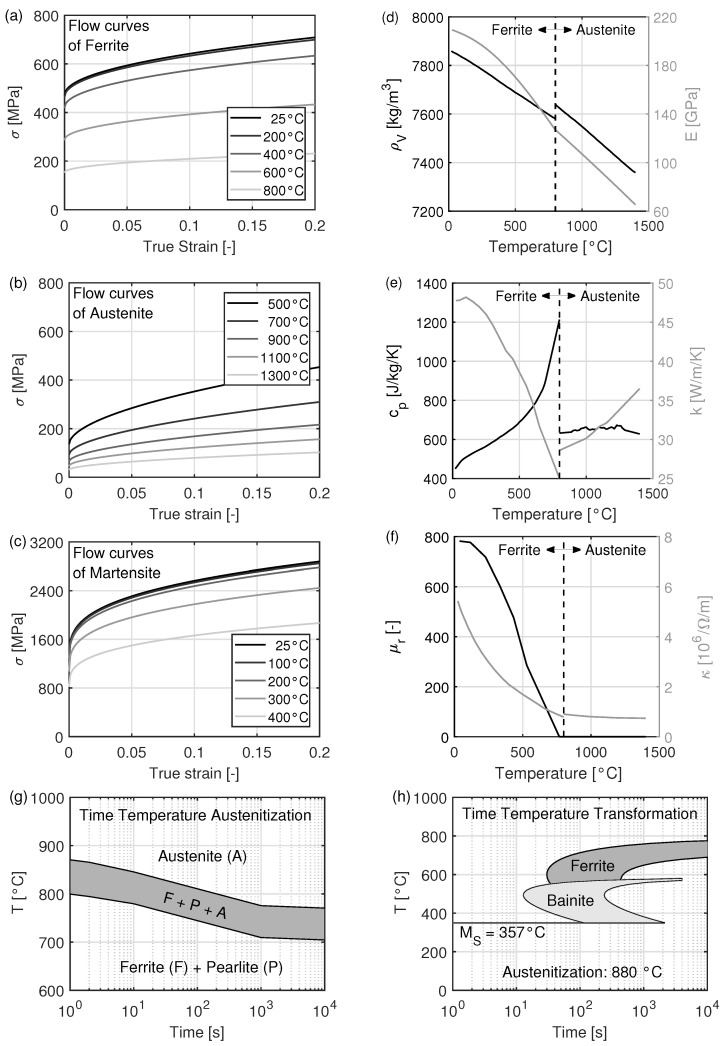
Material data used for the electromagnetic-thermal-mechanical coupled simulation (**a**–**d**) mechanical, (**e**) thermal and (**f**) electrical material data and (**g**,**h**) phase transformation diagrams.

In this work, the boron alloyed heat-treatable steel 34MnB5 has been considered. The properties of this steel, required for the simulation, are shown in Figure 4. The flow curves and the thermal, mechanical and electrical properties, as well as the time-temperature-austenitization (TTA) diagram, were determined experimentally. The time-temperature-transformation (TTT) diagram was calculated based on the chemical composition of the steel with JMatPro^®^ (Matplus GmbH, Wuppertal, Germany).

### 2.3. Finite Element Simulation

The roll forming and the HFI longitudinal tube welding process were simulated by means of FEM with the software MSC Marc/Mentat^®^ 2019 (MSC Software Corporation, Newport Beach, CA, USA). As raw material, a steel band with a width of 110.8 mm, a thickness of 5.8 mm, and a scraping angle of 9.5${}^{\circ }$ was considered. The FE model is described in the following section. Since the simulation of roll forming is well described in literature, e.g., in Han et al. [18,42], the focus is placed on the transition from the roll forming to the welding simulation, as well as on modeling the HFI welding.

**Figure 5:**
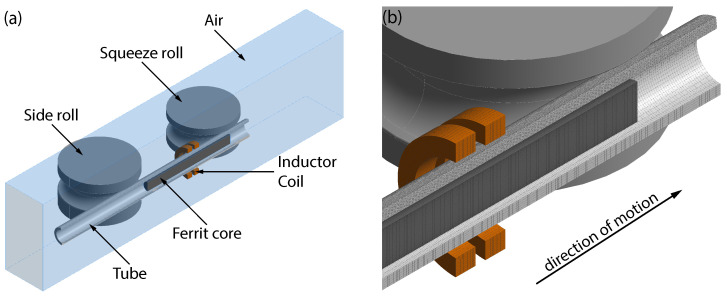
Half model used for the simulation of the HFI welding process (**a**) in full view and (**b**) in detailed view with hexahedron elements.

After completion of the roll forming simulation, the cross section of the open seam tube close behind the fin-pass roll was determined. To create the geometry for the welding simulation, the cross section was extruded to a length of 500 mm. Due to symmetry and in order to reduce the computation time, only half of the tube was considered in the HFI welding simulation, as shown in Figure 5. The coil, a two-winding inductor, was modeled simplified by two rings with an outer diameter of 70 mm and a cross section of 10 mm× 12 mm. The center of the inductor coil was located 95 mm in front of the squeeze roll, which itself was modeled as a rigid body. A current of 2650 A with a frequency of 135 kHz was applied to the inductor coil. In order to enable the simulation of induced currents within the inductor coil, the skin, ring, proximity, and slot effect were considered.

A dual mesh method was used for simulating the HFI welding process. One mesh was generated for calculating the thermal, structural, and phase transformation pass within the tube, while a second mesh was generated for calculating the electromagnetic pass within the coil, the ferrite core, and the surrounding air. The mesh of the air overlapped the mesh of the tube. Due to the dual mesh method with overlapping meshes, the tube can be moved without remeshing. Furthermore, the material properties of the tube are shared with the integration points of the elements used for calculating the electromagnetic pass. This enabled the calculation of the induced currents and the generated heat within the electromagnetic pass based on the actual properties of the tube. The generated heat was passed back to the tube for calculating the temperature field, phase transformations, and the deformation.

With respect to the application of the dual mesh method, the geometry was meshed with linear hexahedron elements with eight nodes and eight integration points. Elements with an edge length of 1.3 mm were used for meshing the tube in the axial direction. Because of the low penetration depth of high-frequency currents, an edge length of 0.125 mm was chosen in the circumferential direction in order to enable a correct simulation of the inductive heating along the band edges. Slightly larger elements with an edge length of 0.750 mm were used for meshing the coil and the ferrite core. The mesh of the surrounding air had an even bigger edge length, which was refined to 0.125 mm in the range of the band edges and the V-shaped opening of the tube in order to calculate the induced currents accurately.

**Figure 6:**
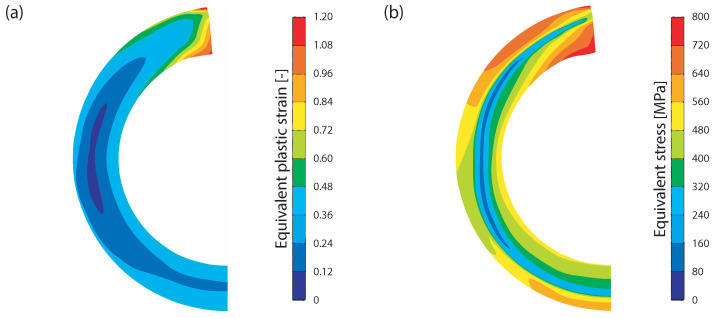
The roll forming simulation calculated (**a**) equivalent plastic strains and (**b**) equivalent stresses, which are mapped onto the open seam tube in the welding simulation.

Prior to the first simulation step, strains and stresses calculated in the roll forming simulation were mapped onto the mesh of the open seam tube. The strains and stresses are shown in Figure 6. Then, the open seam tube was closed at the point of welding by moving the squeeze roll in a lateral direction towards the symmetry plane. Afterward, the inductor coil was powered on and the HFI welding simulation was started. The tube was moved through the squeeze roll at a velocity of 32 m/ min. The time step increment was set to 0.416 mm, so that the tube was moved 250 mm in 600 time steps. This was sufficient to reach a stationary temperature field within the tube. The welding of the band edges was modeled with a thermally-controlled contact condition. This means that the separation of nodes from the symmetry plane was blocked when a node touched the symmetry plane while the nodal temperature was above 1300 ${}^{\circ }C$. In the final simulation step, the squeeze roll was moved back and the cooling process was simulated.

### 2.4. Experimental Validation

In order to validate the simulation, experiments were carried out on a tube welding line of the type RD80 (SMS Meer GmbH, Mönchengladbach, Germany). The temperature on the tube surface was measured close to the band edges with a ratio pyrometer of the type Impac IGAR 6 Advanced (Advanced Energy Industries Inc., Denver, CO, USA). Beside, the force on the squeeze rolls was measured with a load cell of the type LB 216 (Magtrol Inc., Buffalo, NY, USA).

The geometry of the open seam tube was measured prior to welding and after welding on a tactile coordinate measuring machine of the type Leitz PMM-C (Hexagon AB, Stockholm, Sweden). Further, metallographic cross sections were prepared out of the welded tube in order to analyze the microstructure and the hardness of the material within the heat affected zone. The cross sections were etched with a 3% nital solution prior to the microscopy analysis. The latter was performed in brightfield with an optical microscope of the type Axioskop 2 MAT (Carl Zeiss AG, Wetzlar, Germany). The hardness measurements were carried out according to DIN EN ISO 6507-1 with a test load of 4.903 N (=HV 0.5) on a hardness testing machine of the type DuraScan 50 (ZwickRoell, Ulm, Germany).

## 3. Results and Discussion

In the following section, the geometry of the tube, the force on the squeeze rolls, the temperature distribution along the band edges, the phase types after welding, and the hardness distribution within the heat-affected zone obtained by means of FEM are presented and compared to measurements.

### 3.1. Tube Geometry after Roll Forming

**Figure 7:**
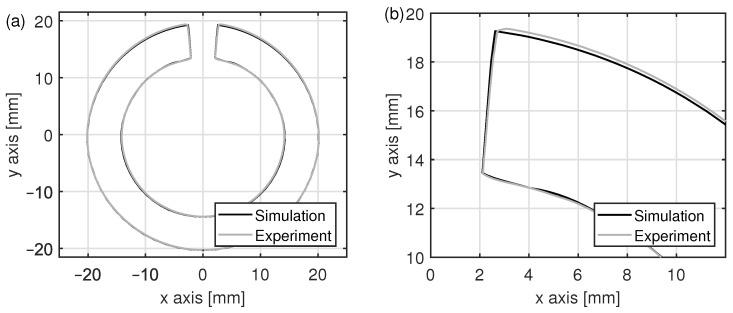
Comparison of simulated and measured tube geometry after roll forming (**a**) in full cross sectional view and (**b**) in detailed view close to the band edge.

The geometry of the open seam tube after roll forming has a large influence on HFI welding. In particular, the temperature distribution at the welding point, the weld bead volume, and the weld seam quality are affected by the geometry of the band edges [31,36]. Thus, it is important to have the correct tube geometry for the welding simulation. In Figure 7, the simulated geometry of the open seam tube after roll forming is compared to the measured geometry. The largest deviation of 0.1 mm occurs in the outer diameter close to the band edge. In this area, the thickness of the steel band of initially 5.8 mm is increased by 0.3 mm during the real forming process, while an increase by 0.2 mm was simulated. A reason for this deviation might be the anisotropy of the material, which was neglected in the simulation. Another reason for the deviation could be variations in the thickness of the steel band and in the roll forming process itself, which have not been considered in the model. However, despite the small deviation close to the band edge, the geometry after roll forming was be predicted very well by means of FEM.

### 3.2. Tube Geometry after HFI Welding

**Figure 8:**
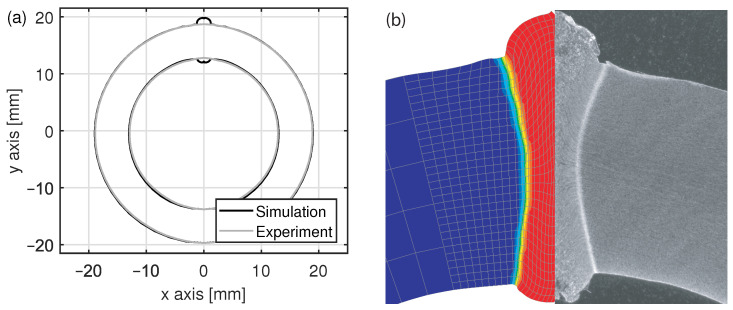
Comparison of (**a**) the cross section of the welded tube and (**b**) the weld bead and heat-affected zone obtained by means of FEM and in the real HFI welding process.

The simulated geometry of the tube including the weld bead is compared to that obtained in the real welding process in Figure 8. Regarding the diameter and the tube wall thickness, the deviation between the measurement and the simulation is less than 0.15 mm. Further, the formation of the weld bead was simulated accurately. This also applies to the hourglass shape of the weld, which is a result of temperature and associated phase transformations. However, the width of the simulated hourglass shape is slightly greater than that obtained in the real welding process.

In the real welding process, the squeezing-out of molten material causes a deflection of segregation lines close to the band edges. In the simulation, these so-called upsetting lines can be visualized by regarding the deformed mesh. As can be seen in Figure 8b, the simulated upsetting lines are about 10${}^{\circ }$ less inclined than those formed in the real welding process. This deviation might be caused by a partial melting of the band edges in the real process, which was not modeled in the simulation. Instead, the flow stress of the material was reduced. However, a sudden drop in viscosity due to melting cannot be modeled this way.

### 3.3. Force on the Squeeze Rolls

The upsetting distance, which is mainly controlled by the position of the squeeze rolls, is another important parameter regarding the quality of the weld seam [43]. However, in this work the resulting force on the squeeze rolls, which is a measure for the welding pressure, was analyzed. By means of FEM, a force of 93 kN on the squeeze rolls was simulated, while the measured force varied between 98 kN and 102 kN. The difference of about 7% is probably due to variations of the yield strength of the steel band, which also might explain the fluctuation in the measurement.

### 3.4. Temperature

**Figure 9:**
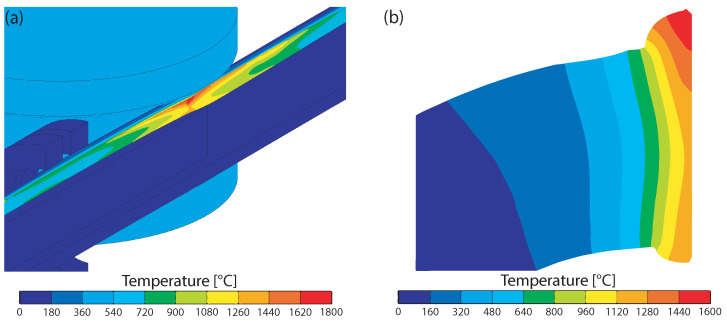
Temperatures obtained by means of FEM (**a**) along the band edge and (**b**) in the cross section at the point where the open seam tube is joined by the squeeze rolls.

Due to the induced current, high temperatures were reached in the band edges, as shown in Figure 9a. The maximum temperature of 1750 ${}^{\circ }C$ occurred where the band edges first touch. This point is located about 15 mm in front of the contact point of the squeeze rolls. The maximum temperature occurred on the outer surface of the tube, while the temperature on the inner surface was about 350 ${}^{\circ }C$ less. However, regarding welding, a uniform temperature over the thickness of the tube would be ideal [30,44]. The temperature distribution in the tube cross section is shown in Figure 9b at the position where the squeeze rolls contact the tube. In this view, it can be seen that the tube was only heated significantly close to the band edges because of the high-frequency current. As is generally known, the yield stress of a material drops with temperature. Thus, only the small area close to the band edges with temperatures above 1000 ${}^{\circ }C$ was deformed plastically during welding, while the rest of the tube was not deformed. Immediately after welding, the temperature in the seam dropped rapidly. This is because the heat was conducted from the hot weld seam to the cold tube.

**Figure 10:**
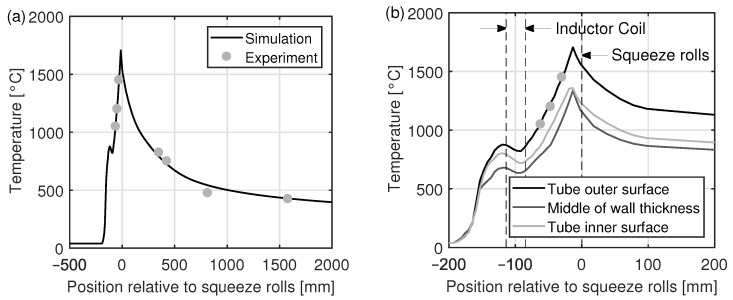
Comparison of the simulated and the measured band edge temperatures (**a**) on the tube outer surface and (**b**) in a small area around the point of welding, with additional representation of the temperatures on the inner tube surface and in the middle of tube wall.

In order to validate the simulation, temperatures were measured in the real welding process on the tube outer surface close to the band edge at three locations upstream and four locations downstream of the point of welding. Since measurements were only possible at locations where the weld seam is accessible, the distance between the measuring points is not equal. This can be seen in Figure 10a, which shows the measured and the simulated temperatures along the band edge. As can be seen, the difference between the measured and the simulated temperatures is less than 100 ${}^{\circ }C$. This applies to the heating and the cooling. In Figure 10b, the simulated temperatures on the inner surface of the tube and in the middle of the tube wall are shown in addition to the temperature on the outer surface of the tube. It can be seen that the heating up of the band edges starts in front of the inductor coil. Close to the inductor coil, the heating is higher at the surface than in the middle of the tube wall. After the inductor coil, a linear heating behavior occurs until the point where the band edges touch. At this point, which is about 15 mm in front of the squeeze rolls, the maximum temperature is reached. After that point, the band edges are cooled down in an exponential manner.

Please notice that in the range of the inductor coil, the tube is slightly cooled. This is because the heating due to the induced current is lower than the cooling due to heat conduction. The reason for the low heat generation is that the magnetic field lines are in parallel to the tube axis within the inductor coil. Thus, only small eddy currents are induced in the tube.

### 3.5. Microstructure Evolution

For the steel used in this work and the simulated heat rate, the transformation from ferrite to austenite begins at a temperature of 795 ${}^{\circ }C$ (Ac${}_{1}$). The transformation is completed when a temperature of 870 ${}^{\circ }C$ (Ac${}_{3}$) is reached. During cooling down, the austenite is transferred to martensite if a temperature below 357 ${}^{\circ }C$ is reached within 20 s. Otherwise, the austenite is transferred back to ferrite. During welding, the Ac${}_{3}$ temperature is exceeded close to the band edges. Since the weld seam quickly cools down afterwards, the austenite is transferred into martensite.

**Figure 11:**
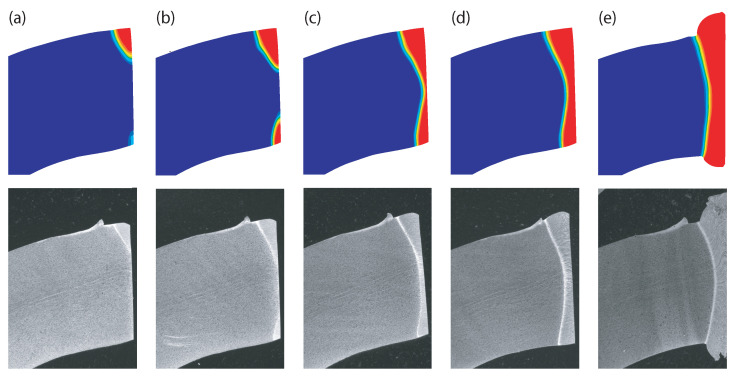
Comparison of the microstructures obtained by means of FEM and by metallographic inspection (**a**) 60mm, (**b**) 50mm, (**c**) 40mm, and (**d**) 30mm in front of the point of welding, as well as (**e**) at the point of welding.

Figure 11 compares the martensite formation determined by means of FEM and by metallographic inspection at different stages of the heating process. Around 60 mm in front of the squeeze rolls, the Ac${}_{3}$ temperature is exceeded in a small area close to the outer tube surface. Hence, a martensite microstructure only exists in this area after cooling down. On the tube inner surface, the temperature necessary for the formation of austenite is reached about 10 mm further. In the middle of the tube wall, the Ac${}_{3}$ is reached even later. Due to this chronological sequence, the martensite microstructure forms an hourglass shape. The hourglass shape, which is typical for HFI welded seams, can first be noticed about 40 mm in front of the squeeze rolls. Until the point of welding is reached, the width of the hourglass shape continuously grows because of heat conduction. Of course, the microstructural evolution depends on temperature. Since the simulated and the measured microstructure are consistent, it is assumed that the temperature curves obtained by means of FEM, which are shown in Figure 10b, are correct as well.

### 3.6. Hardness Distribution

**Figure 12:**
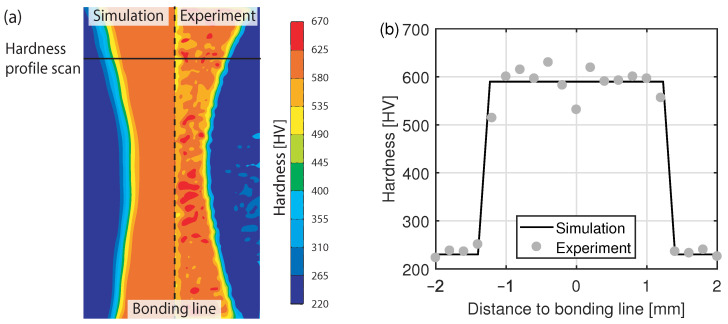
Comparison of the simulated and measured (**a**) hardness distribution within the heat-affected zone and (**b**) hardness profile across the weld seam.

Figure 12 compares the hardness distributions obtained by means of FEM and by measurement. In the simulation, the hardness was calculated based on the resulting microstructure. This means that the averaged hardness values known from measurements were mapped onto the corresponding phases. In regions where phases coexist, the hardness value was calculated on the weighted mean. Hence, only one hardness value per phase exists in the simulation.

In contrast to the simulation, the hardness values measured within the martensite phase fluctuate in a range from 550 to 650 HV. This is because of local differences in chemical composition of the material and segregation lines, which cannot be modeled in the simulation. Furthermore, a hardness decrease was measured in the bonding line. The decrease arises due to reduced carbon content caused by oxidation and diffusion processes in the heated state of the band edges [45]. The diffusion of carbon at interfaces can also be observed in other high temperature processes [46,47]. Since oxidation and diffusion processes were not modeled in the simulation, the local decrease of the hardness in the bonding line cannot be captured by means of FEM. However, in the rest of the heat-affected zone, the hardness values obtained by means of FEM correspond to those obtained by measurement. Furthermore, the width of the martensite phase and the decrease in hardness at the interface between the martensite and the ferrite phase can be predicted correctly.

## 4. Conclusions

In this paper, a fully coupled three-dimensional finite element model for the simulation of a tube manufacturing process consisting of roll forming and HFI welding is presented. By means of a roll forming simulation, the exact band edge geometry and the residual stresses in the open seam tube were determined. These data were then transferred to the welding simulation.

For the coupled electromagnetic-thermal-mechanical HFI welding simulation, a dual mesh method was used. This means that two overlapping meshes were generated to calculate the thermal, structural, and phase transformation pass within the tube, and to calculate the electromagnetic pass within the coil, the ferrite core, and the surrounding air. Due to the dual mesh method with overlapping meshes, the tube could be moved in the simulation without remeshing. Further, the material properties of the tube were automatically shared with the integration points of the overlapping elements used for calculating the electromagnetic pass. This enabled the calculation of the induced currents and the generated heat within the electromagnetic pass based on the actual properties of the tube. In order to mathematically describe the electromagnetic, thermal, and mechanical properties of the tube, nonlinear phase-dependent models were used. Phase transformations were described based on the TTA- and TTT-diagram.

The experimental validation of the simulation results shows that the geometry of the open seam tube, the geometry of the welded tube, the forces on the squeeze rolls, the temperature along the band edges, the hourglass shape of the martensite phase, and the hardness distribution within the heat-affected zone can be predicted accurately by means of FEM. Considering roll forming, the deviation between the measured and simulated tube diameter is less than 0.1 mm. This deviation, caused by a change of the wall thickness, occurs close to the band edges. After welding, the deviation between the measured and the simulated tube diameter is less than 0.15 mm. This also holds for the tube wall thickness. Regarding the temperature during welding, the largest deviation between measurement and simulation is less than 100 ${}^{\circ }C$. This deviation also affects the force on the squeeze rolls, which differs about 7%. Furthermore, the simulated upsetting lines are inclined about 10${}^{\circ }$ less than the lines formed in the real welding process. Small deviations of 40 HV between the simulation and the experiment can also be noticed in the hardness profile. This is because fluctuations of the hardness caused by local differences in chemical composition of the material and segregation lines cannot be modeled. The decrease in hardness of 60 HV in the bonding line cannot be predicted by means of FEM. The decrease is caused by a decarburization of the bonding line due to oxidation and diffusion processes in the heated state of the band edges. However, oxidation and diffusion processes were neglected in the simulation.

Based on the validated finite element model, the complex physical interactions during HFI welding and the influence of manufacturing parameters can now be investigated in a systematic manner. This will help to improve the HFI welding process with regard to the processing of steels with a high carbon equivalent. In order to capture the decarburization of the bonding line, equations that describe the diffusion process must be coupled to the existing model. For that purpose, constitutive equations that describe the carbon transfer from the band edges to the atmosphere as a function of temperature and time must be developed.

## Data Availability

The data generated in this study cannot be shared at this time. They may be available from the corresponding author upon reasonable request.
